# Disseminated *Talaromyces marneffei* infection after renal transplantation: A case report and literature review

**DOI:** 10.3389/fcimb.2023.1115268

**Published:** 2023-02-02

**Authors:** Liang Xu, Xiuxiu Chen, Xuying Yang, Hongtao Jiang, Jianli Wang, Shaowen Chen, Jian Xu

**Affiliations:** ^1^ Department of Organ Transplantation, The Second Affiliated Hospital of Hainan Medical University, Haikou, China; ^2^ The Department of Breast and Thyroid Surgery, The Second Affiliated Hospital of Hainan Medical University, Haikou, China; ^3^ Department of Scientific Affairs, Hugobiotech Co., Ltd., Beijing, China; ^4^ Department of Clinical Laboratory, The Second Affiliated Hospital of Hainan Medical University, Haikou, China

**Keywords:** *Talaromyces marneffei*, renal transplantation, clinical characteristics, antifungal drug, prognosis

## Abstract

We reported a 31-year-old man who received renal transplantation for more than 2 years. He was admitted to our hospital on 9 March 2022 due to intermittent diarrhea accompanied by leukopenia for more than 1 month. The patient successively developed high fever, cough, anemia, weight loss, gastrointestinal bleeding, and liver function impairment. Computed tomography (CT) revealed a slight inflammation in the lower lobes of both lungs, enlargement of the lymph nodes in the retroperitoneal and the root of mesenteric areas, and hepatosplenomegaly. *Talaromyces marneffei* was detected by metagenomics next-generation sequencing (mNGS) in blood and bronchoalveolar lavage fluid, and the pathogen was subsequently verified by blood culture. After endoscopic hemostatic therapy and antifungal therapy with voriconazole and amphotericin B cholesteryl sulfate complex, the patient was successfully discharged. Oral voriconazole was given regularly after discharge. Diarrhea, fever, enlargement of the lymph nodes, and endoscopic evidence of erosion may indicate intestinal *T. marneffei* infection. Although the mortality of *T. marneffei* infection after renal transplantation is very high, timely and effective antifungal therapy with amphotericin B cholesteryl sulfate complex is still expected to improve its prognosis.

## Introduction

*Talaromyces marneffei* (*T. marneffei*), formerly known as *Penicillium marneffei*, is the third most common opportunistic pathogen and the only temperature-dependent dimorphic fungus in the genus *Talaromyces*. This pathogen grows in the filamentous form at 25°C and in the yeast form at 37°C. It is mainly prevalent in Southeast Asia and South China (Ying et al., 2020; Sun et al., 2021; Li et al., 2022; Shi et al., 2022), with a mortality rate of up to one-third (Narayanasamy et al., 2021). In recent years, along with an increase in the frequency of organ transplantation, *T. marneffei* infections have been increasingly reported, and the affected area has a tendency to expand (He et al., 2021; Shen et al., 2022). *T. marneffei* mainly invades *via* the respiratory tract and then spreads to other tissues and organs of the body, including the reticuloendothelial system, skin, and gastrointestinal tract. Cases of *T. marneffei* infection with the digestive tract as original infection site are rare (Zhou et al., 2021). To the best of our knowledge, this is the first case report of *T. marneffei* caused by intestinal infection as the first symptom after renal transplantation. In this paper, we retrospectively analyzed the successful treatment experience of disseminated *T. marneffei* infection *via* digestive tract after renal transplantation. Furthermore, we summarized the clinical characteristics and treatment of *T. marneffei* infection in combination with the relevant literatures.

## Case description

A 31-year-old man living in Hainan, China was admitted to the Second Affiliated Hospital of Hainan Medical University on 9 March 2022 due to low back pain with intermittent diarrhea for more than 1 month. He previously underwent renal transplantation in our hospital on 10 September 2021 due to uremia and was chronically treated with tacrolimus and mycophenolate enteric-coated tablets combined with methylprednisolone for maintenance of immune suppression therapy after surgery. His baseline serum creatinine (Cr) was 300 μmol/L. He had no history of AIDS, and time-zero biopsy of the transplanted kidney revealed no infection. On admission, no lesions were observed in the patient’s skin, and blood routine showed the following results: white blood cell (WBC), 0.71×10^9^/L; hemoglobin (Hb), 88 g/L; blood Cr, 435 μmol/L; procalcitonin (PCT), 17.49 ng/ml; and C-reactive protein (CRP), 463.2 mg/L. Magnetic resonance imaging (MRI) of lumbar vertebra showed Schmorl’s node formation at the upper edge of the L3–5 vertebral body; multiple enlarged lymph nodes were found in the retroperitoneum. On admission, the patient was immediately empirically given piperacillin tazobactam sodium (4.5 g thrice daily) combined with caspofungin (50 mg once daily) for anti-infection treatment, as well as antidiarrheal, leukocyte-elevating treatment, suspension of mycophenolate enteric-coated tablets, and other treatments. The patient’s diarrhea symptoms were relieved, WBC gradually returned to normal, PCT and CRP were decreased within 4 days after hospitalization, but he still had low back pain.

**Figure 1:**
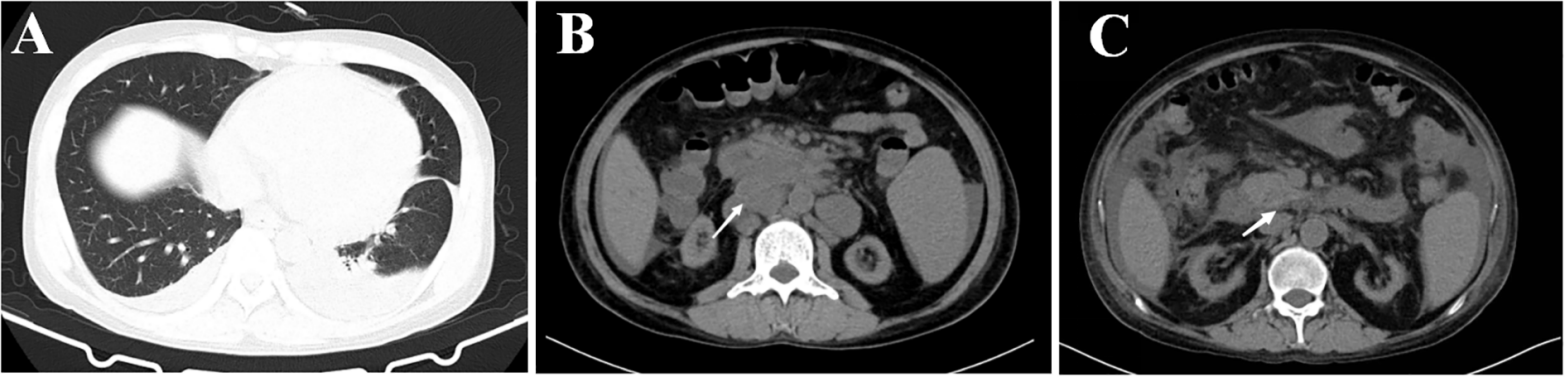
CT image results in lung and abdomen of the patient. **(A)** CT image of lung infection with *Talaromyces marneffei*. **(B)** CT image of abdomen, lymphadenopathy in the retroperitoneum and mesenteric root, and hepatosplenomegaly, the largest of which is 31×25 mm (white arrow). **(C)** CT image of abdomen, lymphadenopathy in the retroperitoneum and mesenteric root, and hepatosplenomegaly are improved, the largest of which becomes normal (white arrow).

**Figure 2:**
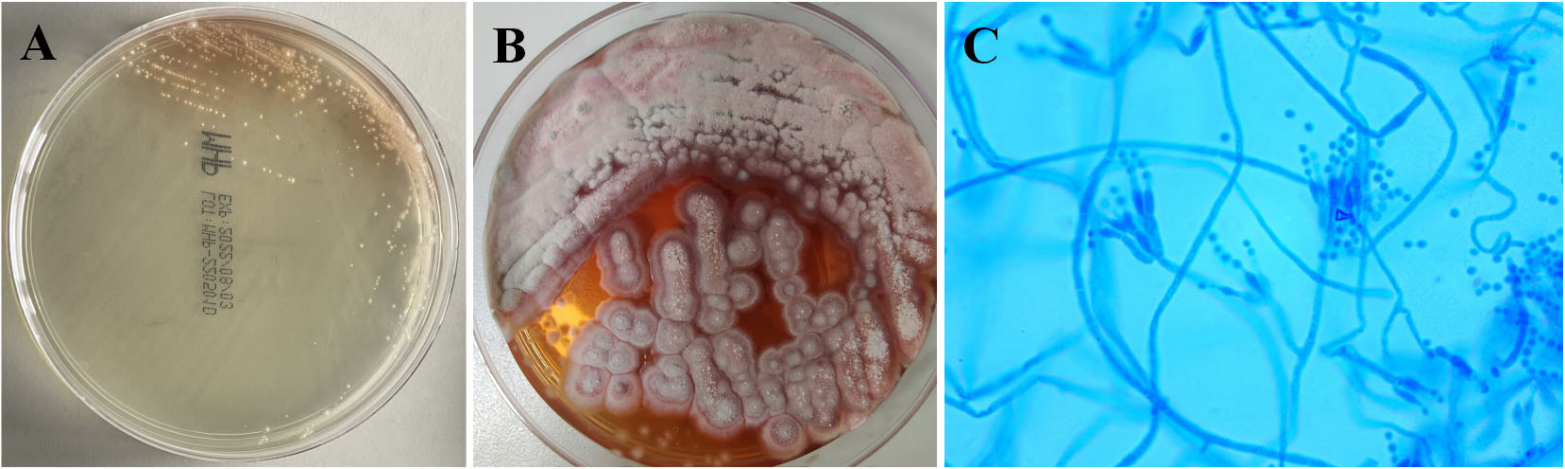
Culture results of this patient. **(A)** Blood culture on Sabouraud’s agar medium plate at 37°C showed yeast phase. **(B)** Blood culture of *Talaromyces marneffei* in the mold phase at 25°C, characteristic red pigment production was observed. **(C)** Lactophenol cotton blue staining from fungal blood culture demonstrating septate hyphae and smooth conidia aloft phialides, which are borne to metulae.

Bone marrow aspiration biopsy (right ilium and sternum) on 14 March 2022 revealed active hyperplasia of the myelogram with significant hyperplasia of the granulocytic lineages, left shift of nuclei, both erythroid lineages and megakaryocytic hyperplasia, and visible platelets. On 16 March 2022, the patient presented with a sudden onset of high fever (39.2°C) accompanied by a small amount of melena. Blood biochemical tests on 9 March 2022 revealed the following results: alanine aminotransferase (ALT), 37 U/L; aspartate aminotransferase (AST), 124 U/L; blood Cr, 469 μmol/L; PCT, 4.7 ng/ml; and CRP, 106 mg/L. The 1,3-β-glucan test revealed negative results (55.51 pg/ml). CD4+ T-cell count was 48/μl. Fecal occult blood test was weakly positive. CT on 17 March 2022 showed a little inflammation in the lower lobes of both lungs, lymphadenopathy in the retroperitoneum and mesenteric root, and hepatosplenomegaly (**Figures 1A, B**). The antibiotic regimen was changed to meropenem (1 g thrice daily), caspofungin (50 mg once daily), and ganciclovir (0.25 g twice daily). The patient still had irregular fever, and the low back pain was aggravated than before. The liver and kidney function were further deteriorated, with an ALT level of 310 U/L, an AST level of 1686 U/L, a blood Cr level of 613 μmol/L, a PCT level of 17.45 ng/ml, and a CRP level of 463.2 mg/L. On 21 March 2022, simultaneous mNGS (Hugobiotech, Beijing) using blood and bronchoalveolar lavage fluid (BALF) was performed: DNA was extracted using QIAamp DNA Micro Kit (QIAGEN, Hilden), and DNA libraries were built using QIAseq™ Ultralow Input Library Kit (QIAGEN, Hilden) according to the manufacturer’s instructions. Qubit (Thermo Fisher, MA) and Agilent 2100 Bioanalyzer (Agilent Technologies, Palo Alto) were applied for quality evaluation of the libraries. Nextseq 550Dx platform (Illumina, San Diego) was used for mNGS detection (~10 M 75-bp single-end reads after sequencing). After removing short, low-quality, low-complexity, and human host reads, the remaining reads were aligned to Microbial Genome Databases (http://ftp.ncbi.nlm.nih.gov/genomes/) using BWA. *T. marneffei* was detected in both blood and BALF samples, and the number of unique sequences of *T. marneffei* in blood and BALF was 15,499 and 4373, respectively. The coverage of *T. marneffei* in blood and BALF detected by mNGS was 3.63% and 1.07%, respectively. The pathogen was subsequently confirmed by blood culture on 28 March 2022 (**Figures 2A–C**). Considering the compromised liver and kidney function of the patient, the anti-infective regimen was adjusted to voriconazole (200 mg twice daily) and meropenem (1 g thrice daily) on 21 March 2022, accompanied by methylprednisolone (80 mg/day) for immune maintenance treatment; anti-rejection drugs was stopped. The blood concentration of voriconazole was maintained at 2–5 μg/ml. After antifungal therapy, liver and kidney function and infection indicators of the patient were gradually improved, but diarrhea symptoms were aggravated than before, with watery stools about 500 ml/day.

On 11 April 2022, the patient had a shock after a sudden red bloody stool of 800 ml. Emergency endoscopic hemostasis and blood transfusion therapy were performed immediately. Gastroscopy revealed chronic non-atrophic gastritis. Colonoscopy revealed multiple ulcers in the colon, including bleeding from an ulcer surface in the ileocecal junction, which was stopped after cauterization and titanium clip hemostasis. Therefore, we excluded gastrointestinal bleeding caused by glucocorticoids, and the bleeding was considered associated with *T. marneffei* infection. On 21 April 2022, the patient received enteroscopy again due to red bloody stool of 600 ml; the bleeding point was still located in the ileocecal region, the same as the first bleeding. After blood transfusion, endoscopic hemostasis, and other treatment measures, the patient’s gastrointestinal bleeding temporarily stopped. Unfortunately, repeated bleeding with 600 ml of red bloody stool occurred on 4 May 2022. Blood transfusion, endoscopic hemostasis, and other treatment measures were once more taken. Given that repeated endoscopic hemostasis was ineffective, voriconazole could only control parts of symptoms, and his liver function returned to normal, amphotericin B cholesteryl sulfate complex (4 mg/kg) was given for anti-infection on 2 May 2022. Gratifyingly, the patient’s fever, diarrhea, gastrointestinal bleeding, and other clinical symptoms were gradually improved, and the infection indicators also gradually returned to normal.

**Figure 3:**
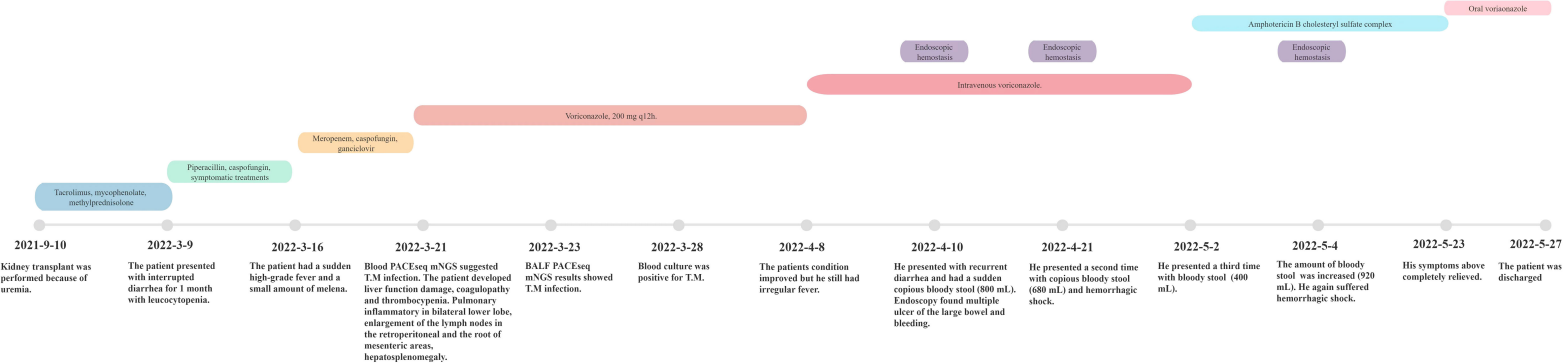
Important clinical information of the recipient and treatment timeline after renal transplantation.

After 3 weeks of antifungal therapy with amphotericin B cholesteryl sulfate complex, the patient was discharged on 27 May 2022, with serum creatinine maintained at 250 μmol/L. The patient continued maintenance therapy with voriconazole 200 mg twice daily. Methylprednisolone (4 mg) was maintained for anti-rejection therapy after discharge for 3 months, and tacrolimus was then added with a concentration of about 3 ng/ml; the blood Cr fluctuated between 280 and 327 μmol/L. After 7 months of follow-up, repeat abdominal CT showed resolution of the retroperitoneum and mesenteric root lymphadenopathy (**Figure 1C**), and no adverse events have been found. Important clinical information and treatment timelines for this patient during hospitalization are presented in **Figure 3**.

## Discussion

*T. marneffei* was first isolated by Capponi et al. (1956) at the Pasteur Institute in 1956 from the liver of the Vietnamese bamboo mouse, but no formal description has been published. In 1959, laboratory personnel inoculated *T. marneffei* when the finger was pricked, causing local nodular lesions and ipsilateral axillary lymph node enlargement, and the fungus was named *Penicillium marneffei*. The first natural human infection of *T. marneffei* was reported in 1973 in an American patient with Hodgkin’s disease who was living in Southeast Asia (Cao et al., 2019). In 2011, the RNA polymerase II largest subunit gene was sequenced and the phylogenetic relationship was analyzed; it was renamed *T. marneffei*, and the deep mycoses caused by *T. marneffei* were referred to as talaromycosis (Samson et al., 2011). *T. marneffei* infection can involve multiple organ systems, such as lung, skin, bone marrow, digestive system, and disseminated infections (Ding et al., 2021); the vast majority of the patients presented with fever, respiratory symptoms, anemia, skin lesions, hepatosplenomegaly, weight loss, and lymphadenopathy (He et al., 2021). The mortality rate of disseminated talaromycosis is high, reaching 80%–100% in patients without timely diagnosis and antifungal treatment; despite receiving antifungal treatment, its mortality rate is still as high as 30% (Li et al., 2021). At present, most scholars usually believe that yeast phase conidia of *T. marneffei* are first inhaled into the lungs, and then phagocytosed by macrophages and spread throughout the body *via* the blood circulation (Castro-Lainez et al., 2018); disseminated *T. marneffei* infection with the digestive tract as the first infection site in renal transplant recipients has not been reported.

In our case, the patient is living in Hainan for a long time and had no history of contact with bamboo mouse. The zero time biopsy of transplant kidney showed uninfected with *T. marneffei*, and the donor-derived infection could be excluded completely. The patient presented with low back pain with diarrhea as the first symptom and gradually developed pneumonia (mild pulmonary imaging changes) and gastrointestinal bleeding, which eventually improved after antifungal therapy. Imaging findings suggested retroperitoneal lymphadenopathy, and endoscopy also revealed multiple ulcers and bleeding in the colon. Therefore, we suspect that the patient had digestive tract bleeding caused by *T. marneffei* infection. Previous studies have shown that the clinical characteristics of intestinal *T. marneffei* infection included CD4 T cells < 50/μl, fever, abdominal pain, diarrhea, abdominal distension chronic gastrointestinal symptoms, abdominal lymphadenopathy (considered as indirect evidence of enterogenous infection), endoscopically confirmed erosions or ulcers, and biopsy of colonic tissue samples (Xie et al., 2022). Combined with this patient’s condition, we suspect that the route of infection in this patient was digestive tract. Negative stool cultures alone cannot exclude enteric infection with *T. marneffei* due to the low load of fungi in the intestinal lumen (Pan et al., 2020). Fortunately, the early diagnosis and prompt treatment of this patient are due to mNGS technology (Zhang et al., 2020), which provides strong support for clinicians in the diagnosis of rare pathogen infections (Wilson Michael et al., 2014), even as an indication to determine whether to discontinue antifungal drugs (Xu et al., 2022). It should be noted that we have no direct evidence that the route of dissemination was *via* the digestive tract; the intestinal wall biopsy was not taken for this patient due to concerns that it might aggravate gastrointestinal bleeding.

Currently, there are rare cases of *T. marneffei* infection after kidney transplantation. We searched literatures in the Web of Science and PubMed databases based on “*Talaromyces marneffei*”, “*Penicillium marneffei*”, “kidney transplantation”, and “renal transplantation”, which had been published up to October 2022. A total of 10 officially published papers reporting 11 cases of *T. marneffei* infection after kidney transplantation were retrieved (Wang et al., 2003; Chan et al., 2004; Lin et al., 2010; Hart et al., 2012; Peng et al., 2017; Lang et al., 2020; Vergidis et al., 2021; Gupta et al., 2022; Li et al., 2022; Xing et al., 2022) (**Table 1**). The above 11 recipients were summarized in order to enhance the cognition of *T. marneffei* infection after renal transplantation and improve the prognosis. Of the 11 recipients, 7 were men and 4 were women; the age ranged from 34 to 67 years, with a median age of 47 years. Time from kidney transplantation to discovery of *T. marneffei* infection varied from 3 months to 11 years, with a median of 48 months. Six of the 11 patients had fever as the first symptom, 4 had cough with sputum, and only 1 had dry cough as the main symptom. Two patients had abdominal pain and one of them was complicated with diarrhea. One patient each had symptoms including subcutaneous nodules, scalp swelling, bone pain, and bladder irritation. At the same time, we found that the organs that were affected the most were the lungs (five cases), followed by skin (two cases), bone (two cases), and kidney (two cases). One of two patients with abdominal cavity involvement presented with mesenteric lymphadenopathy. Respiratory tract infection was identified in only 5 of 11 patients, and no gastrointestinal infection was reported. Among the 11 patients, 7 had positive blood cultures for *T. marneffei*, 2 had positive tissue cultures, and 2 were diagnosed by mNGS. Almost all patients were treated with reduced anti-rejection drugs, mainly because antifungal drugs (amphotericin B, liposomal amphotericin B, voriconazole, posaconazole, and fluconazole) had a side effect of increasing the concentration of tacrolimus. Two of the 11 patients died after treatment with voriconazole, including one due to resuscitation (as a result of rapid atrial fibrillation) failure and the other due to deterioration of the disease and abandonment of treatment after discharge. Six patients were cured with amphotericin B or liposomal amphotericin B combined with itraconazole. One patient each was cured with itraconazole, voriconazole, or posaconazole. The duration of treatment varied from 1 to 12 months, with a median of 9 months. Combined with our case, we found that fever was the most common initial symptoms of *T. marneffei* infection, whereas the specific clinical symptoms varied, which is mainly related to the infection sites. Hart et al. (2012) also reported a case of disseminated *T. marneffei* infection with diarrhea as the initial symptom in 2012. However, the patient was finally diagnosed with cytomegalovirus enteritis causing sigmoid perforation; fungal infection in digestive tract was not considered.

**Table 1:** Clinical information of *Talaromyces marneffei* infection after kidney transplantation.

Author	Region	Gender/age	Onset time (days)	Clinical presentation	Radiological imaging/Endoscopy	Infection route	Infection site	Diagnostic methods	The use of immunosuppressive agents	Treatment	Disease duration (months)	Prognosis
Wang et al. (2003)	Taiwan, China	M/47	33	Fever, dry cough, poor appetite, weight loss, skin lesions, subcutaneous masses.	Osteomyelitis	NA	Skin, bone	Cultures of the blood and debrided specimens	Reduced Tac dosage	L-AmB (2 mg/kg, 28 days), itraconazole (400 mg/day).	12	Survival
Chan et al. (2004)	Hong Kong, China	M/38	9	Fever, chills and rigor, central abdominal pain, cough for 1 week.	Pulmonary infiltrates, enlarged mesenteric lymph nodes	NA	Lung, bone, lymph nodes	Blood and bone marrow cultures, lymph node biopsy	Reduced Tac dosage, withheld MMF	AmB (30 days, cumulative dose of 0.75 g), itraconazole (200 mg/day).	9	Survival
Lin et al. (2010)	Taiwan, China	F/42	9	Pain in her left hip.	Osteolytic lesion on the left pelvic brim near acetabulum	NA	Bone	0 mg	NA	L-AmB (2 mg/kg, 21 days), oral itraconazole (200 mg per day, 8 months)	8	Survival
Hart et al. (2012)	Australian	M/67	48	Abdominal pain and diarrhea.	Normal	NA	Abdominal	Blood and peritoneal fluid cultures	Reduced Tac dosage	L-AmB (3 mg/kg) for 14 days, oral itraconazole.	8	Survival
Peng et al. (2017)	China	M/51	13	Fever and worsening renal functions.	Increased renal volume, hyperechoic and hypoechoic lesions in renal allograft	Respiratory tract	Kidney	Blood and biopsy of renal allograft	Withheld Tac and MP, change to CSA, continued MMF	L-AmB (0.4 mg/kg/day, 14 days), followed by itraconazole.	1	Survival
Vergidis (Vergidis et al., 2021)	England	F/53	132	Cough with expectoration, night sweats, chills, decreased exercise.	Chest x-ray: left upper lobe mass; CT: mediastinal lymphadenopathy	Respiratory tract	Lung	Fine needle aspiration of the involved lymph nodes and lung mass	Reduced Tac dosage, withheld MMF	AmB (2 weeks), followed by itraconazole (12 months)	12	Survival
Lang et al. (2020)	China	M/34	48	A 4-month history of weakness and poor appetite, occasional nonproductive cough with hemoptysis.	CT: new patchy consolidation	Respiratory tract	Lung	NGS of BALF, fungal culture of BALF and blood	Reduced Tac and MMF dosage	Oral posaconazole at the dose of 10 ml (400 mg) bid.	6	Survival
Gupta et al. (2022)	India	F/41	84	Swelling on the right side of the scalp, pain and blurring of vision in the right eye.	CT: right-sided subcutaneous nodule; MRI: lytic lesion in right calvarium	NA	Subcutaneous infection	Fine-needle aspiration of the scalp lesion	NA	Itraconazole 200 mg twice daily.	10	Survival
Li et al. (2022)	China	F/47	132	Fever, frequent urination, and discomfort.	Ultrasonography: 5 mm separation of the transplanted kidney collecting system	Respiratory tract	Kidney	Cultures of peripheral blood and clean midstream urine	Reduced Tac, MMF, MP	Voriconazole (0.2 g, every 12 h), itraconazole capsules (200 mg/day).	NA	Survival
Xing et al. (2022)	China	M/61	4	A repeated cough, expectoration, intermittent fever.	CT: multiple nodules, patchy high-density shadows, and highdensity glass	NA	Lung	NGS of BALF	Reduced Tac	Voriconazole (0.2 g, every 12 h).	8	Died
Xing et al. (2022)	China	M/55	3	A repeated fever and shortness of breath after activity.	CT: ground glass-like shadows, a nodule in right lung	NA	Lung	NGS of peripheral blood	Tac and MMF discontinued, changed to MP 80 mg/day	Voriconazole	NA	Died

L-AMB, liposomal amphotericin B; AmB, amphotericin B; MMF, mycophenolate mofetil; NA, not available; CSA, cyclosporine; Tac, tacrolimus; MP, methylprednisolone; Itra, itraconazole; NGS, next-generation sequencing; Pred, methylpred; CT, computed tomography; BALF, bronchoalveolar lavage fluid; MRI, magnetic resonance imaging.

The therapeutic efficacy of voriconazole was unsatisfactory, and amphotericin B or liposomal amphotericin B remains the first choice. Xing et al. (2022) concluded that high-dose glucocorticoids should be used with caution in patients with *T. marneffei* infection in the transplant state. In our case, we believe that early antifungal therapy is critical. Based on the fact that glucocorticoids not only control fever symptoms but also maintain immunosuppression, we believe that the rational use of glucocorticoids does not affect prognosis, at least in our case.

Reducing the dose of anti-rejection drugs was mentioned in 9 of the 11 patients, but no specific rationale was detailed. In this case, we also reduced the dose of tacrolimus and MMF drugs. One reason is that both tacrolimus and MMF can inhibit T-cell activity or proliferation (Ferreira et al., 2020; Chen et al., 2021). Previous studies indicated that the incidence of *T. marneffei* infection was lower in patients with higher CD4+ T-cell lymphocyte counts. The probability of *T. marneffei* infection was 1.65% when the CD4+ T-cell count ≥ 200 cells/μl, 14.89% when the CD4+ T-cell count < 200 cells/μl, and 28.06% when the CD4+ T-cell count < 50 cells/μl (Chen et al., 2017). Thus, the reduction or depletion of CD4+ T cells in recipients with *T. marneffei* infection is considered not conducive to infection control. Macrophages are unable to activate and kill bacteria (immune escape) if CD4+ T cells are reduced or depleted, resulting in massive macropinocytosis and massive fungal reproduction, systemic disseminated infection caused by macrophages through lymphatic and blood circulation may happen. Significant macrophage increase occurs in the reticuloendothelial system, manifesting as enlargement of the liver, spleen, and lymph nodes, resulting in focal necrosis of organs (Dong et al., 2019). Based on the clinical symptoms and the prognosis of this patient, a regimen of complete discontinuation of anti-rejection drugs and maintenance of immunosuppression with only steroids turned out to be safe and feasible in recipients with serious disseminated *T. marneffei* infections. However, clinicians still need to carefully decide whether such serious infection will endanger the patient’s life, or whether it is controlled effectively without the use of immunosuppressive agents.

The mortality rate of gastrointestinal bleeding caused by *T. marneffei* infection in HIV patients is extremely high, and the successful management experience is not much (Cui et al., 2022). Due to the lack of guidelines for *T. marneffei* infection in organ transplant patients, as well as the concurrent severe impairment of liver and kidney function in our patient, voriconazole was used for induction therapy as recommended by the Centers for Disease Control and Prevention (Masur et al., 2014). However, symptoms of the patient were only partially resolved, and gastrointestinal bleeding persisted. In this case, when the recipient suffered from hemorrhagic shock caused by repeated gastrointestinal bleeding, the recipient was treated with two massive blood transfusions and two endoscopic hemostatic treatments with temporary success. Our team believes that surgical treatment alone is difficult to achieve effective therapeutic effects for recurrent gastrointestinal bleeding caused by *T. marneffei*, because it may still lead to postoperative intestinal anastomoses or bleeding anywhere in the intestine. Therefore, after his liver and kidney function improved, we decided to use antifungal therapy with amphotericin B cholesteryl sulfate complex (Thompson et al., 2021). Since there was no itraconazole in our center, consolidation therapy with voriconazole was still performed after induction therapy with amphotericin B cholesteryl sulfate complex and the treatment was successful.

## Conclusions

In summary, recipients who have received immunosuppressive agents for a long time after renal transplantation, especially those who have lived or traveled in *T. marneffei*-endemic areas, should be alert to *T. marneffei* infection for unexplained symptoms, such as high fever, cough and expectoration, hepatosplenomegaly, lymphadenopathy, osteolytic damage, and even gastrointestinal bleeding. As shown in our case, hemorrhagic shock due to gastrointestinal bleeding is often fatal, and how to treat patients to the greatest extent through measures such as hemostasis, blood transfusion, and antifungal therapy requires clinicians to develop a more individualized treatment strategy based on available resources.

## Data availability statement

The datasets presented in this study can be found in online repositories. The names of the repository/repositories and accession number(s) can be found in the article/supplementary material.

## Ethics statement

Written informed consent was obtained from the individual(s) for the publication of any potentially identifiable images or data included in this article.

## Author contributions

JW designed the paper. LX, XC, and XY drafted and revised the manuscript. LX, HJ, and JX carried out the clinical care and management of the patient. SC did the fungal identification tests. All authors contributed to the article and approved the submitted version.
